# Automated microseismic event location using Master-Event Waveform Stacking

**DOI:** 10.1038/srep25744

**Published:** 2016-05-17

**Authors:** Francesco Grigoli, Simone Cesca, Lars Krieger, Marius Kriegerowski, Sergio Gammaldi, Josef Horalek, Enrico Priolo, Torsten Dahm

**Affiliations:** 1 National Institute of Oceanography and Experimental Geophysics (OGS), Trieste, Italy; 2 GFZ German Research Centre for Geosciences, Potsdam, Germany; 3 School of Physical Sciences, University of Adelaide, Australia; 4 Department of Physics, University of Naples, Italy; 5 Czech Academy of Sciences, Prague, Czech Republic

## Abstract

Accurate and automated locations of microseismic events are desirable for many seismological and industrial applications. The analysis of microseismicity is particularly challenging because of weak seismic signals with low signal-to-noise ratio. Traditional location approaches rely on automated picking, based on individual seismograms, and make no use of the coherency information between signals at different stations. This strong limitation has been overcome by full-waveform location methods, which exploit the coherency of waveforms at different stations and improve the location robustness even in presence of noise. However, the performance of these methods strongly depend on the accuracy of the adopted velocity model, which is often quite rough; inaccurate models result in large location errors. We present an improved waveform stacking location method based on source-specific station corrections. Our method inherits the advantages of full-waveform location methods while strongly mitigating the dependency on the accuracy of the velocity model. With this approach the influence of an inaccurate velocity model on the results is restricted to the estimation of travel times solely within the seismogenic volume, but not for the entire source-receiver path. We finally successfully applied our new method to a realistic synthetic dataset as well as real data.

The increasing number of microseismic monitoring networks for both seismological and industrial applications has led to an exponential growth of available microseismicity data in the last decade. These data typically contain a large number of weak seismic events, whose waveforms are often highly noise contaminated. Standard location routines based on automated phase picking, identification, and association tend to fail when dealing with such noisy data^1^ or when simultaneous events occur^2^. Therefore, an improved, fully automated, and noise robust procedure must be established in order to obtain high precision location for weak events. The main systematic limitation of traditional event location techniques is that the automated event identification is most commonly performed individually on single seismograms, thereby making little or no use of the coherency information between waveforms recorded at different stations^3^. The increasing interest on microseismic monitoring operations, particularly for oil and gas reservoirs applications, motivated the development of new location methods based on waveform stacking techniques commonly used in reflection seismics^2^. These methods exploit the coherence of the waveforms recorded at different stations and do not require any automated phase picking, identification and association procedure. The Source Scanning Algorithm (SSA) developed by Kao and Shan^4^,^5^ represented the first attempt to use the signal coherency information for automated detection and location of earthquakes. In later years, several modified versions of this pioneer algorithm have been proposed and used for different applications, including monitoring of natural^6^,^7^,^8^ and induced seismicity^1^,^9^,^10^, volcano seismology^11^,^12^, and landslides monitoring^13^. The main advantage of this class of methods is that location results are robust even in presence of noisy waveforms^1^,^9^,^10^. However, like any other absolute location method, their location performance still strongly depends on the accuracy of the a priori seismic velocity model^14^,^15^. The adoption of inaccurate models may lead to large errors and uncertainties of the location results, which affect the output of further geological and geophysical analysis (e.g. estimation of source mechanism, event magnitude, etc.). The negative effect of velocity model inaccuracies on location results can be mitigated using relative location methods, which are generally based on source-specific station correction terms^14^ or on the differential travel times for seismic sources that are next to one another^16^. In case of a localized cluster of seismic events these techniques can heavily improve the relative location accuracy^14^, but in the end they are also based on automatic picking procedures, therefore they inherently suffer from the same problems affecting classical location approaches based on phase picking. The first attempt to include stations corrections in backprojection methods was proposed in 2007^17^. This method is based on the use of seismic array at teleseismic distances, and it was applied for the rupture imaging of the 2004 December 26th, Sumatra Earthquake (M_*w*_ 9.2).

Here we propose an improved automated location method suitable for microseismic monitoring applications. This technique combines the waveform stacking approach with the main features of relative location methods. Thus our method inherits the advantages of full-waveform location methods while sensibly reducing its dependency on the accuracy of the velocity model. In this study we follow a master event approach^18^,^19^. A master event is genereally characterized by a highly reliable hypocentral location and a high signal-to-noise ratio of its associated waveforms at all (or almost all) the stations of the network. As a first step high quality seismic data from large seismic events are used to evaluate the effect of earth inhomogeneities on travel times (i.e. determine station residuals). Thereafter the observed travel times for smaller events with an hypocenter close to the master event are adjusted accordingly. Residuals are computed for a specific ray path and are valid for all events within the first Fresnel zone of the master event^20^. By applying this adjustment we are able to successfully locate small scale seismic events in a fully automated way, even when the available velocity model is poorly known or when the topographic effects are very strong (e.g. volcanic areas).

In the following, we introduce and describe the theory of our algorithm. Thereafter, we prove the feasibility of the concept by analysing a synthetic data set, which resembles a realistic scenario. Finally, we assess the performance of the master-event waveform stacking event (re-)location method, by applying it to 119 weak events from a seismic swarm in NW Bohemia, Czech Republic.

## Theoretical concept of Waveform stacking event location

### Classical Waveform Stacking location

**Figure 1:**
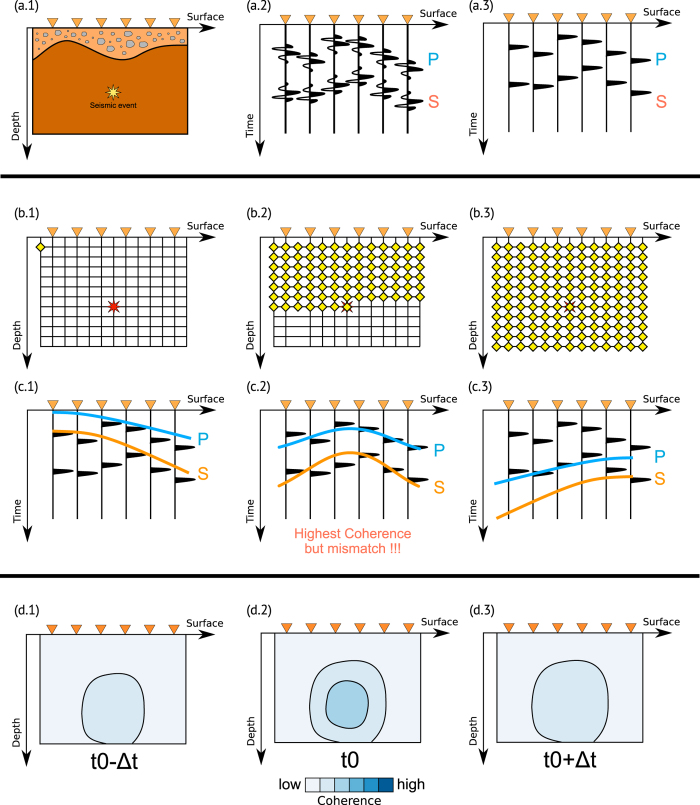
(Panel a) Seismic event recorded by a line of receiver deployed on the surface (a.1), raw (a.2) and processed (a.3) traces. (Panel b) waveform stacking is performed by scanning different source locations (b.1, b.2, b.3) and different origin times (c.1, c.2, c.3)). (Panel c) The output of the location process is a multidimensional coherence matrix (d.1, d.2, d.3) whose maximum corresponds with the hypocenter and the origin time t0 of the seismic event (d.2).

We briefly introduce the classical waveform stacking (WS) location method, and without loss of generality we can follow here the scheme proposed by Grigoli *et al*.^7^,^9^. We consider an ideal 2D case for this description, as the extension to 3D is trivial. The processing steps are conceptually sketched in Fig. 1.

Let us suppose to have a linear array of *n* receivers deployed at the surface and a seismic event occurring at a certain location (Fig. 1a.1). To remove the effect of the source radiation pattern from the original seismograms (Fig. 1a.2) we first calculate non-negative stacking functions from the recorded waveforms. Optimal stacking functions need to be sensitive to the *P-* and *S-*wave onsets. Several definitions for these stacking functions exist^2^, they are generally processed seismic waveforms showing spike-like high amplitude signals corresponding to the main seismic phase onsets (Fig. 1a.3). The theoretical traveltimes for *P-* and *S-*waves at station *i* (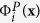 and 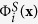 respectively) for a potential source location **x** can be computed from the source-receiver geometry and the a priori velocity model. We then define the reduced *P-* and *S-* traveltimes (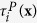 and 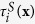 respectively) as:


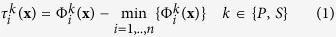


from these reduced traveltime functions we infer the likelihood of a potential source location by stacking (i.e. delay and sum) waveforms along theoretical *P-* and S-wave arrival times and then estimating their coherence (see Fig. 1c.1–c.3):


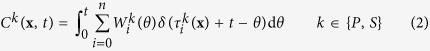


where *δ* is the Dirac’s delta and 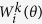 is a *P-* or *S-*wave stacking function related to the *i*-th station. Waveform normalization is generally required to remove the attenuation effect due to the geometrical spreading: in this way we avoid that stations close to the source dominate the stacking. The coherences for the two seismic phases are then evaluated together in terms of the general coherence function


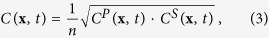


which is bounded between 0 (no coherence) and 1 (perfect coherence for both P and S phases). This function is a measure of the coherence of the seismic wavefield for different source locations and origin times, estimated along both the P and S theoretical travel time surfaces.

The value of *C*(**x**, *t*) is determined by the event origin time and the source location. From a grid search for these parameters we then obtain a map of the coherence in space and time (Fig. 1d.1–d.3); the hypocentral location **x**_0_ and origin time *t*_0_ are defined in correspondence to the highest overall coherence:


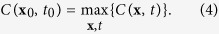


The location process described in this paper is performed using two different stacking functions based on the Short-Term-Average/Long-Term-Average (STA/LTA) traces, which are designed to enhance both P and S first onsets. For P phases, we use the STA/LTA of the squared vertical trace^7^ (we call this trace P-STA/LTA), whereas for the S phases we use the STA/LTA of the maximum eigenvalue of the instantaneous covariance matrix computed using the horizontal components traces^7^ (we call this trace S-STA/LTA). Location and origin time uncertainties can be estimated using different methods: randomly perturbing the STA/LTA parameters and relocating the same event several times^9^, fitting the coherence function with a multidimensional Gaussian distribution^11^, or transforming the coherence function in a probability density function^3^.

### Master-event Waveform Stacking location

**Figure 2:**
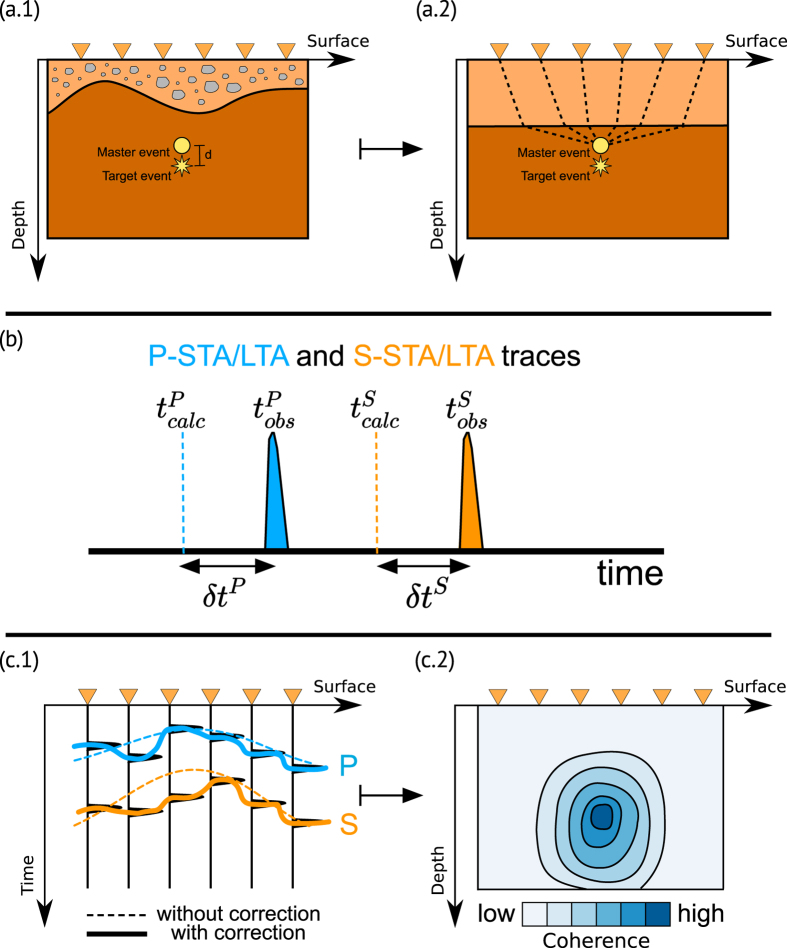
Panel (a.1) shows the master event (its location is supposed to be known) and the target event to be located. We then calculate the theoretical traveltimes of the master event, for a given velocity model (panel a.2) and we compute the correction term for each station (panel b). Panel (c.1) represents the master-event WS process using the corrected traveltimes (continuous lines). With respect to the standard WS the coherence obtained by using this approach is higher (panel c.2).

The negative effect of a poorly known velocity model can be mitigated by extending the standard WS location with typical features of relative location methods. We use information from well located large events with small location uncertainties to evaluate the effect of unmodeled earth heterogeneities on travel-times (i.e. determine station residuals) and to adjust the observed traveltimes for smaller events in their local vicinity^21^. We will refer to such large reference events as “master” events, while we call “target” the respective small events, whose location will improve by using information from the master Fig. 2a.1). The master-event location is based on the assumption that errors in the velocity model are equal for observations from two repetitive events at the same location. This assumption is usually still valid if the inter-event distances are negligible compared to the event-station distances. Consequently, this method can only be applied to seismic events within a restricted volume in the neighbourhood of the master event. If the hypocentral separation between the master event and a target event is small, not only compared to the event-station distance but also to the scale length of the velocity heterogeneity, the ray paths between the two hypocenters and a common station are sufficiently similar^16^.

A master event is characterized by a highly reliable hypocentral location and a high signal-to-noise ratio of its associated waveforms; Therefore, master events should be generally selected from the set of events of largest magnitudes. The most important condition which a master event must satisfy is that they need to be recorded with a reasonable signal-to-noise ratio at many (ideally all) stations, so that reliable station corrections can be obtained for most of the receivers. Since a good master event can be found late after the beginning of a seismic sequence, the precise relocation using the master-event technique would then require anyway the reprocessing of the catalogue upon the selection of the master event.

We can now reformulate the WS location (equations (1)–(4), 14, 18, 19) following a master event approach. We assume a single master event *M*, which has been accurately located at **x**^*M*^. We are interested in the location of a target event *T*, at **x**^*T*^ in the vicinity of **x**^*M*^. The method can be applied if the inter-event distances are much smaller than the event-station distances and if the target event lies within the first Fresnel zone of the master event^20^. This last condition is possibly too strict, but it is here given as a rule of thumb to estimate the maximum inter-event distance between the master and the target event. Out of the Fresnel zone the performance of the location method starts to decrease. However, it is important to note that the performance is often still very good out of the Fresnel zone, when compared to a standard waveform stacking location, and that the Fresnel zone limit should be not considered as a sharp border between correct and wrong results.

The travel-time correction for data recorded at station *i* include the difference between the observed and the calculated reduced traveltimes (see equation (1)), they are evaluated for each station and seismic phase individually:


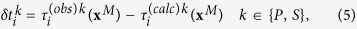


where 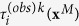 and 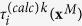 are the observed and theoretical reduced travel-times respectively. We add this correction term 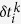 to equation (2), which then becomes:


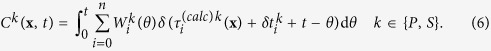


For localized clusters of events these correction terms often lead to a dramatic improvement in location results, the effect is schematically shown in Fig. 2. Correction terms are evaluated at each station, as the difference between the theoretical traveltimes and the maximum peak of the P-STA/LTA and S-STA/LTA, as shown in Fig. 2 (panel b). Waveform coherence strongly increase by adding these correction terms to the theoretical traveltimes used for the stacking process (Fig. 2, panel c).

However, this approach becomes less effective when events become more spatially distributed; a single set of station corrections cannot effectively describe the full effect of the 3D variations^14^. Therefore, we modify the travel-time correction approach by considering several master events, which uniformly sample the focal region. We now have *N*_*M*_ available master events within the cluster of seismic events. We again start by estimating the travel-time correction terms, but now for all master events 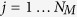 with locations 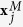:


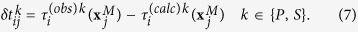


Traveltime corrections are applied within a spherical region of radius *r*, containing the whole seismicity cluster. Outside this region such corrections are not applied. We then calculate a space dependent general travel-time correction term from a weighted mean of this set of individual travel-time corrections:


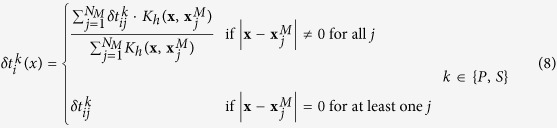


with *K*_*h*_ defined in the following way:


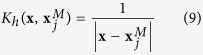


where 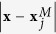 is the Euclidean distance between **x** and 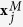. We can optionally use other kernel smoothers (e.g. Triangular, Gaussian etc) to avoid sudden changes in the function 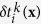. The corrected coherence can be now be written equivalent to eq. (6):


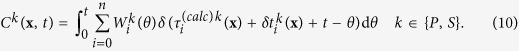


In case of an event distribution in several clusters, which would have to be handled independently with classical methods, our algorithm can still be applied using source-specific station correction terms, as proposed by Richards-Dinger and Shearer^14^.

## The 2008 NW Bohemia seismic swarm: synthetic and real dataset

**Figure 3:**
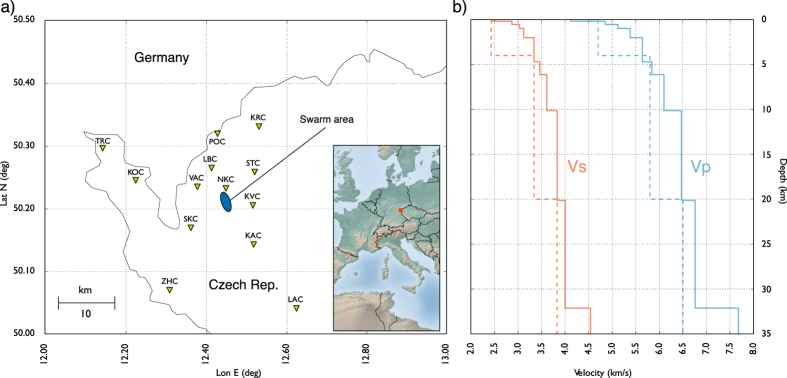
(Panel a) North-West Bohemia Seismic Network (WEBNET). (Panel b) Velocity model proposed by Horalek *et al*.^31^ (continuous line) and a simplified velocity model (dashed line). We used the simplified model (dashed lines) to locate synthetic events and the detailed model^31^ (continuous lines) to locate the real ones. The map has been generated using MATPLOTLIB^34^.

The NW Bohemia and Vogtland regions, located at the border between Czech Republic and Germany, have been repeatedly interested in the past decades by crustal non-volcanic earthquake swarms. Seismic activity in this region is documented in terms of macroseismic observations since the 19^*th*^ century, and instrumentally recorded since 1985, when the strongest earthquake (ML 4.6)^22^ occurred. Since then, about 90% of the earthquake swarms clustered beneath the village Novy Kostel. Single swarms typically consist of several hundreds to thousands of earthquakes with magnitudes mostly below ML < 3.5. Since 1990, major earthquake swarms occurred in 1997, 2000, 2008 and 2011^23^ and were recorded by the WEst Bohemia seismic NETwork (WEBNET)^24^,^25^,^26^. This network consists of 13 three component seismic stations (Fig. 3, panel a) with a inter-station distance ranging from 5 to 25 km with most of the stations (about 8 over 13) within 10 km from the swarming area (Fig. 3, panel a). In May 2014, a magnitude ML 4.5 earthquake occurred in the Novy Kostel epicentral region at about 8 km depth, followed by an aftershock sequence, and differing from the swarm-like seismicity. The review publication by Fisher *et al*.^27^ provides a broad overview of recent works targeting the NW Bohemia earthquake swarms. Swarms episodes are typically characterized by spatially clustered locations^25^,^28^. The geometry of hypocenters and the orientation of focal mechanisms suggest swarms correspond to the activation of a complex distribution of sub-planar surfaces with almost NS striking and different steep dip angles, suggesting the repeated activation of a complex system of faults in correspondence to fluid transfer. Swarm seismicity is clustered at shallow depth, with most energy released between 6 and 13 km^25^,^26^. Few events are located deeper, and none shallower, what has been attributed to the presence of a stiff impermeable body, which presence is confirmed by active seismic studies^29^ and local earthquake tomography^30^. The 2008 NW Bohemia swarm took place during the month of October and only lasted about 4 weeks, with a fast release of seismic moment compared to previous swarms. According to Fisher **et al**.^28^, 25,000 events in the magnitude range of 0.5 < ML < 3.8 were detected. Station inter-distances of the inner-network (i.e. the 8 closest stations to the swarming area) are in the range of 5–10 km. The seismicity and network geometries are optimally suited to test the proposed location technique. We limit our test to a reduced case, considering the last 2 days of the 2008 NW Bohemia swarm (12-13/10/2008). In this time frame, 115 events were reported with magnitude ML 1.0–2.5. Since for this dataset highly reliable locations are also available, we used this data to test our location method. For this applications the volume containing the whole cluster consists of 151 × 219 × 151 grid-points with a grid-spacing of 0.04 km, the origin of the coordinate system is placed at (Lat(deg), Lon(deg), Z(km) = 50.1696, 12.4098, 0.0) and the lower left corner of the grid is shifted (with respect this point) by 17.040 km in the East direction, 17.040 km in the North direction and 6.0 km in depth.

### Synthetic data results

**Figure 4:**
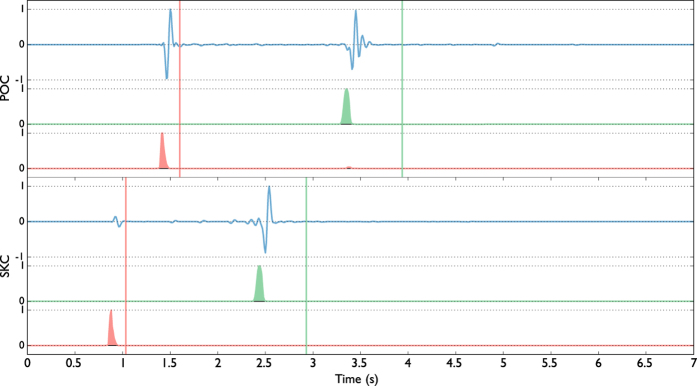
Synthetic waveforms (Z-comp, blue traces), P-wave (red trace) and S-wave (green trace) characteristic functions and theoretical arrival times of the P (red v-line) and S (green v-line) phases obtained using a wrong velocity model (Fig. 3b, dashed lines). The red and green traces represent the P-STA/LTA and the S-STA/LTA respectively (short-time-window = 0.1 s and long-time-window = 0.2 s).

**Figure 5:**
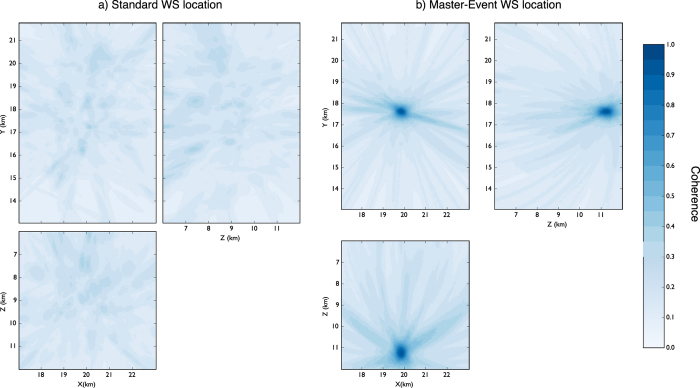
Coherence matrices of a sample event obtained using the standard WS (**a**) and the master-event WS (**b**) location methods. The coherence matrix XY is obtained by projecting, for each point (X, Y), its maximum along the Z directions (coherence matrices XZ and YZ are obtained in a similar way). Coherence values are represented in colour scale. The reference point (X, Y) = (0, 0) corresponds with the point (lat(deg) = 50.1696; lon(deg) = 12.4098) in the geographical coordinates system.

**Figure 6:**
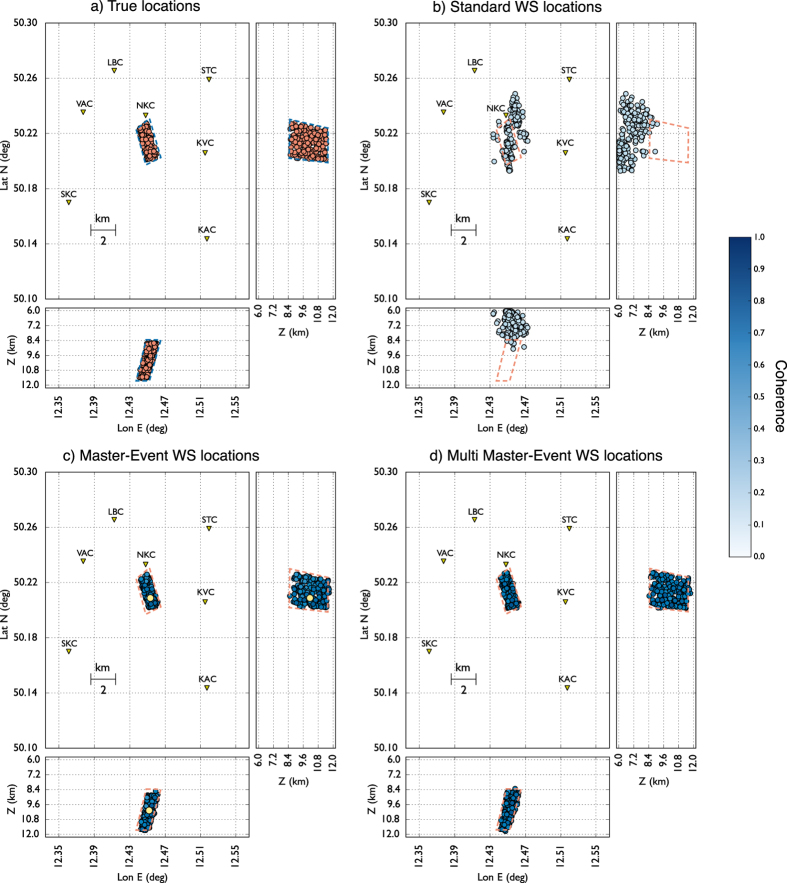
(Panel a) Synthetic events, waveforms were generated using the velocity model proposed by Horalek *et al*.^31^ (Fig. 3b, continuous lines) Location results obtained using the standard (Panel b), master-event (Panel c) and multi master-event (Panel d) waveform stacking location using a wrong velocity model (Fig. 3b, dashed lines). Coherence values are represented in colour scale. The Yellow dot in (**c**) represents the master event, while for panel (d) we selected 9 master-events which homogeneously sample the cluster.

**Figure 7:**
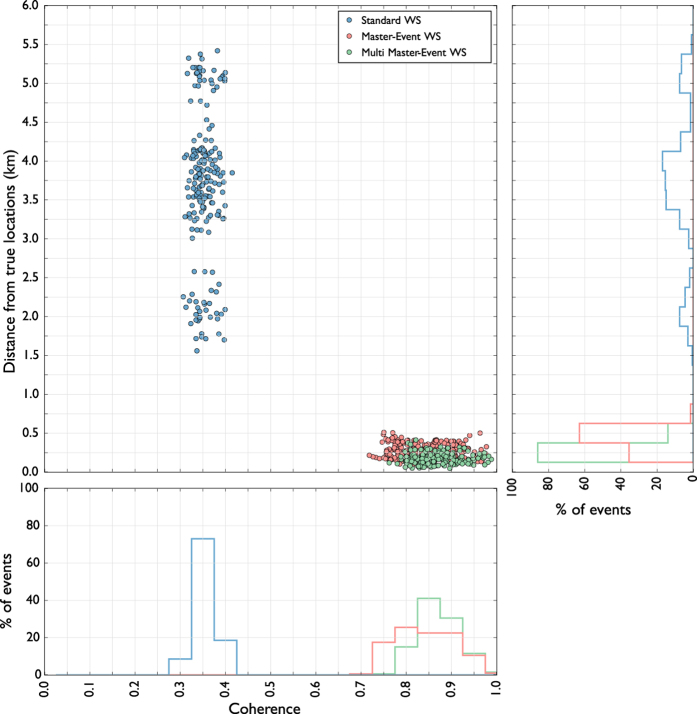
Coherence against distance from true locations and event distribution. Standard WS results are represented in blue, master-event WS results in red and multi master-event WS results in green.

We firstly tested the performance of our location method with a synthetic dataset composed of 200 seismic events using the same configuration of the WEBNET seismic network. In order to make a more realistic experiment, we generated a synthetic dataset with the same characteristics of the real one. We first generated a synthetic catalogue, imposing realistic conditions on hypocentral distribution, magnitude and mechanisms of the seismic events. For each event, we generate then the corresponding synthetic waveforms, which are computed for a detailed velocity model^31^, using a reflectivity algorithm^32^. The obtained waveforms well resemble recorded signals, as it is shown in the comparison of synthetic waveforms and real data for one reference event (Fig. 4). Figure 4 shows a set of waveforms related to two different stations (POC and SKC) of the network. The blue traces represent normalized velocity seismograms (Z component), while the red and the green traces are, respectively, the normalized P-STA/LTA and S-STA/LTA functions (see Grigoli *et al*.^7^ for further details) we used for the stacking process. We used a short-time window length of 0.1 s, while the long time window is twice longer. As discussed before, the performance of standard WS location is affected by our limited knowledge of the seismic velocity structure. To test the effectiveness of the new approach we applied the location process using a simplified velocity model, different from the one used to generate synthetic data. The effect of such approximation can be clearly seen in Fig. 4, where the computed arrival times do not corresponds with those observed on waveforms. The two velocity models are compared in Fig. 3(b). We apply both the standard and master-event WS location methods to the whole synthetic dataset. Figure 5 shows, for a selected target event, coherence matrices obtained with both methods. In this test we first calculated traveltimes using the simplified velocity model, then we added the master-event time corrections, computed by using the equation 5. Similarly to the sketch shown in Fig. 2c.1), the better performance of the master-event WS location methods is proven by the higher coherence values obtained during the location process (compare the panels a and b in Fig. 5). We also further improve the Master-Event WS location method by using several master events homogeneously distributed within the cluster (we selected 8 master events at edge of the cluster and one located at its centre). Results are summarized in Fig. 6, where we show the distribution of the synthetic events (panel a), the locations obtained using the standard WS (panel b), the Master-Event WS (panel c) and the Multi Master-Event WS (panel d) location method. The yellow dot in Fig. 6 (panel c) denotes the position of the master event, while for the multiple master event WS (Fig. 6, panel d) we used 9 master-events which homogeneously sample the cluster. Although coherence matrices of the master-event WS and the multi master-event WS show high similarity (since they are almost identical we did not show it in Fig. 5), the multi master-event WS method brings to slightly better results (Fig. 7). However from the analysis of the Fig. 7, both methods, with respect to the standard one, lead to a strong improvement both in term of location error and coherence. Most of the events located with the standard WS method show a coherence within the range of 0.3–0.4, while using the new approaches coherence values increased to 0.8–0.9. In a similar way, the location error reduced from 3.0–4.0 km to 0.2–0.5 km. These results confirm the high potentiality of this approach in microseismic monitoring operations.

### Real data results

**Figure 8:**
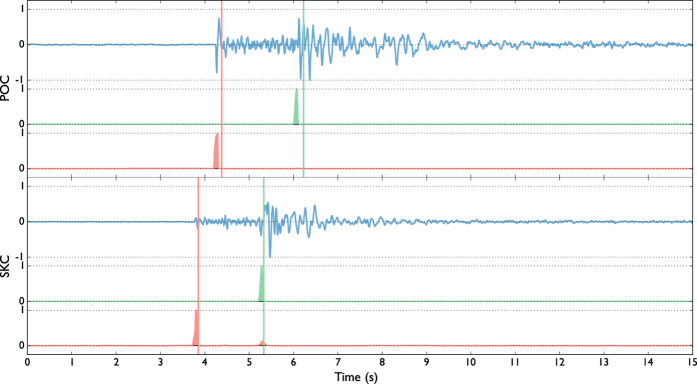
Example of recorded waveforms (Z-comp, blue traces), P-wave (red trace) and S-wave (green trace) characteristic functions and theoretical arrival times of the P (red v-line) and S (green v-line) phases obtained using the best 1D velocity model in the area (Fig. 3b, continuous lines)). The red and green traces represent the P-STA/LTA and the S-STA/LTA respectively (short-time-window = 0.1 s and long-time-window = 0.2 s).

**Figure 9:**
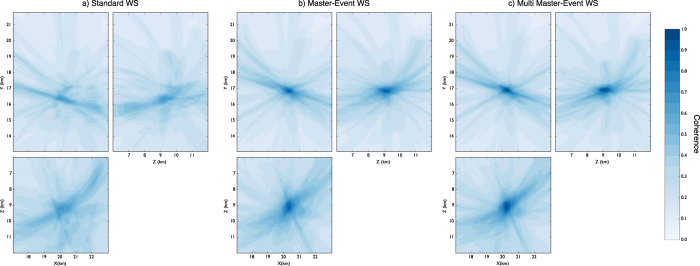
Coherence matrices of a sample event obtained using the standard WS (**a**), the master-event WS (**b**) and multi master-event WS (**c**) location methods. The coherence matrix XY is obtained by projecting, for each point (X, Y), its maximum along the Z directions (coherence matrices XZ and YZ are obtained in a similar way). Coherence values are represented in colour scale. The reference point (X, Y) = (0, 0) corresponds with the point (lat(deg) = 50.1696; lon(deg) = 12.4098) in the geographical coordinates system.

**Figure 10:**
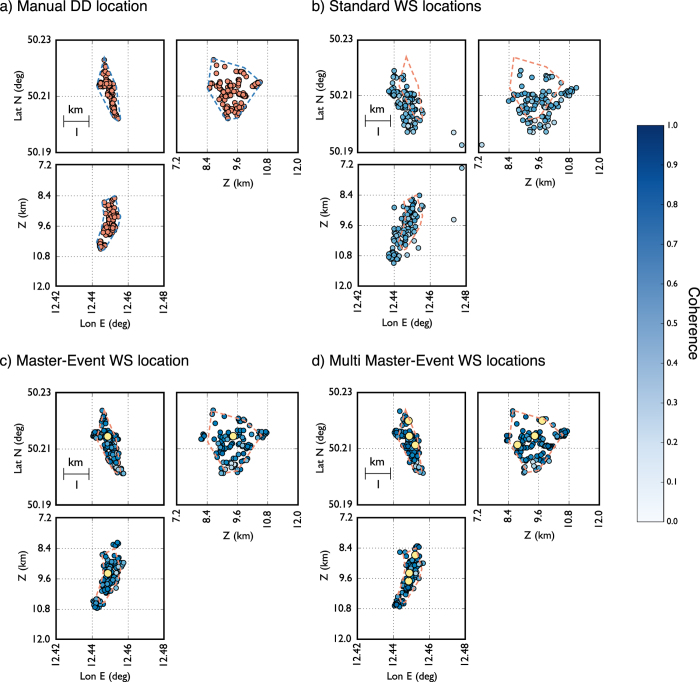
(Panel a) Event located using manual picking and refined using double-difference algorithm. Location results obtained using the standard (Panel b), master-event (Panel c) and multi master-event (Panel d) waveform stacking location using the 1D velocity model prosed by Horalek *et al*.^31^ (Fig. 3b, continuous lines). Coherence values are represented in colour scale. Yellow dots represent the master events.

**Figure 11:**
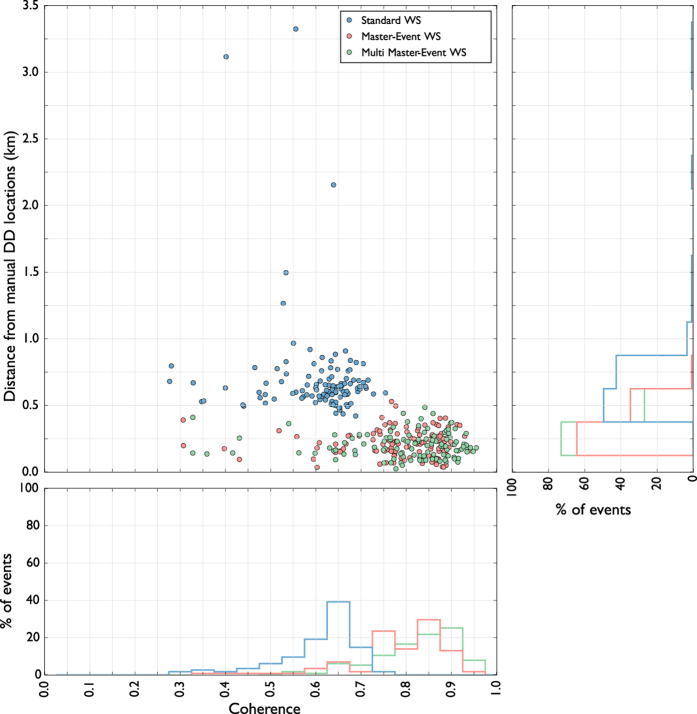
Coherence against distance from DD locations taken as reference. Standard WS results are represented in blue, master-event WS results in red and multi master-event WS results in green.

Upon the successful test with synthetic data, we apply the location method to real data. For this scope, we used a dataset from days 12/10 and 13/10 of the 2008 NW Bohemia swarm. According to the WEBNET catalogue^24^, the dataset is composed of 115 earthquakes with local magnitude ML > 1.0. We use data from the 8 stations of the WEBNET seismic network, which are the closest stations to the swarming area. In analogy with the synthetic case, Fig. 8 shows, for the same pair of stations, the normalized raw seismograms (Z component, blue traces) and the normalized P-STA/LTA and S-STA/LTA functions (the red and green traces respectively) we used for the stacking process. Also in this case we used a short-time window length of 0.1 s, while the long time window is twice longer. Seismic event location has been performed by using the velocity model proposed by Horalek *et al*.^31^, which is one of the most detailed 1D models of the area. The 3 largest events (those with ML> = 2.0) were chosen as master events, and we perform both single master-event and the multi-master event locations. The master events have been selected from the WEBNET catalogue^33^ and have been located using classical location methods based on manual picking procedures. Furthermore these events satisfy the master event requirements described in the previous sections. This is an optimal case to test our location method since all the events of the dataset are clustered within a small volume (within 2 km radius from the centre of the cluster). To asses the quality of our location we compare our results with reliable and high precision Double-Difference locations^33^ and further compare with results obtained by using the standard WS method. For this dataset we estimated a Fresnel zone of about 1.0–1.5 km containing almost the entire cluster. For a selected target event (2008 October 13^*th*^ 09:24:08 UTC ML = 2.0), Fig. 9 shows the comparison of coherence matrices obtained using the standard WS (panel a), the master event WS (panel b) and multi master-event WS location method (panel c). Like in the synthetic case, the ME and MME are characterized by higher coherence values with a more focused maximum. Location results for the whole dataset are summarized in Fig. 10, where we show the DD locations based on manual picking procedure and refined by using cross-correlation (panel a), the standard WS locations (panel b) and the master-event and the multi master-event WS locations (panel b and c respectively). The analysis of the Fig. 11 confirm the better performance of the new methods also with a real dataset. An improvement, both in term of location error and coherence, is clearly visible. Most of the events located with the standard WS method show a coherence within the range of 0.6–0.7, while using the new approaches coherence values increased to 0.8–0.9. Similarly, the absolute distance from the DD locations, taken as reference, reduced from 0.5–3.0 km to 0.2–0.5 km. These results show that these new methods can be successfully applied to locate microseismic event, even when detailed velocity models are not available.

## Discussion and Conclusion

We developed a methodology to locate seismic events using waveform stacking and traveltime corrections estimated from one or more master-events. The proposed method is fully automatic, picking free, noise robust and less dependent on the velocity model, than standard waveform stacking methods. Nowadays, there is a general agreement concerning that WS location methods perform better than the traditional absolute location methods based on phase picking^3^,^9^,^10^; several studies found that WS methods lead to more stable results, especially when outliers are present in the data due to noisy stations for which reliable picks cannot be obtained^10^. Furthermore, for microseismic networks with detailed 3D velocity information the available waveform stacking based methods are preferable to pick-based methods due to their noise robustness and higher automation, better facilitating rapid high-quality results for interpretation^10^. However, a poor knowledge of the velocity model affects both standard location procedures as well as waveform stacking methods. To overcome this limitation, we improved a former WS method by introducing a master-event approach at microseismic scale. This method requires at least one seismic event characterized by a highly reliable hypocentral location (i.e. the master event). The most important condition that a master event must satisfy is that they need to be recorded with a reasonable signal-to-noise ratio at many (ideally all) stations, so that reliable station corrections can be obtained for most of the receivers. After testing the location approach with synthetic data, we successfully applied the master-event and the multi master-event WS methods to locate 115 seismic event (*Ml* > 1.0) recorded by the WEst Bohemia seismic NETwork (WEBNET)^24^. For quality assessment purposes we further compared our results with high precision DD locations^33^. Both comparisons confirm the significant improvement in location accuracy achieved by the new location method. We located more than 65% of the synthetic events within 0.25 km from the reference locations, against the 0.75 km error for a standard WS location. Locations of real seismic events by the master-event and multi-master event methods are more spatially clustered than those obtained using the standard WS approach. Best results (especially concerning a precise depth estimation) are obtained by using the multi master-event method; this is likely due to the fact that reference traveltime corrections are evaluated for master events located at different depths. Furthermore the multi-master event location allows to apply this waveform stacking approach to seismicity clusters showing larger spatial extension without loss of performance. The advantages and disadvantages of the proposed method with respect to the other ones are summarized in Table 1. To conclude, the high level of automation and the high-resolution locations, comparable to DD location methods, which characterize the new approaches make them well suitable for microseismic monitoring operations. In particular, they may be suitable for monitoring induced seismicity close to industrial sites (e.g. Oil and Gas applications), where these methods can be easily implemented within a real time system to facilitate a rapid results interpretation and decision making process.

## Additional Information

**How to cite this article**: Grigoli, F. *et al*. Automated microseismic event location using Master-Event Waveform Stacking. *Sci. Rep.* **6**, 25744; doi: 10.1038/srep25744 (2016).

## Figures and Tables

**Table 1:** Advantages and disadvantages of different automated seismic event location methods.

**Method**	**Advantages**	**Disadvantages**
Standard waveform stacking location method	Fully automated Noise robust	Need accurate velocity models Computationally expensive
Double Difference location method	Less dependent on velocity model Computationally faster	Require similar waveforms Problems with noisy waveforms
Master event waveform stacking location method	Fully automated Noise robust Less dependent on velocity model	Computationally expensive
